# Greater efficiency of the double-bending method compared with conventional endoscopic submucosal dissection for the treatment of submucosal tumors of the gastric fundus

**DOI:** 10.1007/s00464-025-12522-6

**Published:** 2026-02-03

**Authors:** Guihua Duan, Tian He, Linting Xun, Liyue Zheng, Zhengji Song, Ping Wan, Yu Zhang, Ruochang Li, Huanan Duan, Qianfei Ning, Qiang Guo, Zan Zuo

**Affiliations:** Department of Gastroenterology, The First People’s Hospital of Yunnan Province, The Affiliated Hospital of Kunming University of Science and Technology, Kunmin, 650032 China

**Keywords:** Double-bending method, Endoscopic submucosal dissection, Gastric fundus, Submucosal tumors

## Abstract

**Background and aim:**

In clinical practice, endoscopic resection of submucosal tumors (SMTs) in the gastric fundus is extremely difficult. The aim of our study was to compare the efficiency and safety of the double-bending method (DBM) and conventional endoscopic submucosal dissection (ESD) for the treatment of SMTs in the gastric fundus.

**Methods:**

The clinical data of 190 patients who underwent resection of SMTs in the gastric fundus were retrospectively analyzed.

**Results:**

In all, 190 patients with an average age of 53 years were included in this study: 100 patients in the DBM-ESD group and 90 patients in the ESD group. The average operation time was 54.2 min in the DBM-ESD group and 101.6 min in the ESD group (*P* < 0.001), which indicates that the resection speed was significantly faster in the DBM-ESD group. Subgroup analysis revealed that the larger the tumor volume was, the greater the difference in operation time between the two groups. No significant difference was observed in intraoperative or postoperative complications.

**Conclusions:**

DBM-ESD can be safely and effectively performed to resect SMTs in the gastric fundus and is especially suitable for patients with very large tumor diameters.

**Supplementary Information:**

The online version contains supplementary material available at 10.1007/s00464-025-12522-6.

With the growing use of digestive endoscopy, an increasing number of gastric submucosal tumors can be identified. Gastric submucosal tumors (SMTs), including gastrointestinal stromal tumors (GISTs), leiomyomas, neuroendocrine tumors (NETs), lipomas, and neurogenic tumors, originate from the muscularis mucosae, submucosa, or muscularis propria [1]. Stomach SMTs are predominantly benign, and only a handful of GISTs and NETs have malignant potential. Most gastric SMTs are discovered via gastroscopy, and their diagnosis and malignancy evaluation are often difficult. Diagnosis depends on postoperative pathology; therefore, early resection of SMTs is highly important. Endoscopic surgery has become an important treatment for gastric submucosal tumors (SMTs) [2, 3]. Endoscopic submucosal excavation (ESE) and endoscopic full-thickness resection (EFR) are two common therapeutic methods, both of which are derived from endoscopic submucosal dissection (ESD) [4]. Notably, the difficulty in treating submucosal tumors varies according to their location; for example, endoscopic resection of lesions in the fornix is extremely difficult in clinical practice. The gastric wall at the fornix is thin, and incision closures via gastroscopy are difficult; moreover, the resection procedure requires a U-turn of the distal tip of the gastroscope at this location, which increases the difficulty and potential complications of the treatment [5]. To resolve this problem, we developed the double-bending method (DBM) for use during endoscopic treatment by modifying conventional techniques (see Fig. 1 for details). In this study, the efficiency and safety of DBM-ESD and ESD were retrospectively analyzed in 190 patients with SMTs in the gastric fundus. The results lay a foundation for the application of this method in the clinic.

**Fig. 1:**
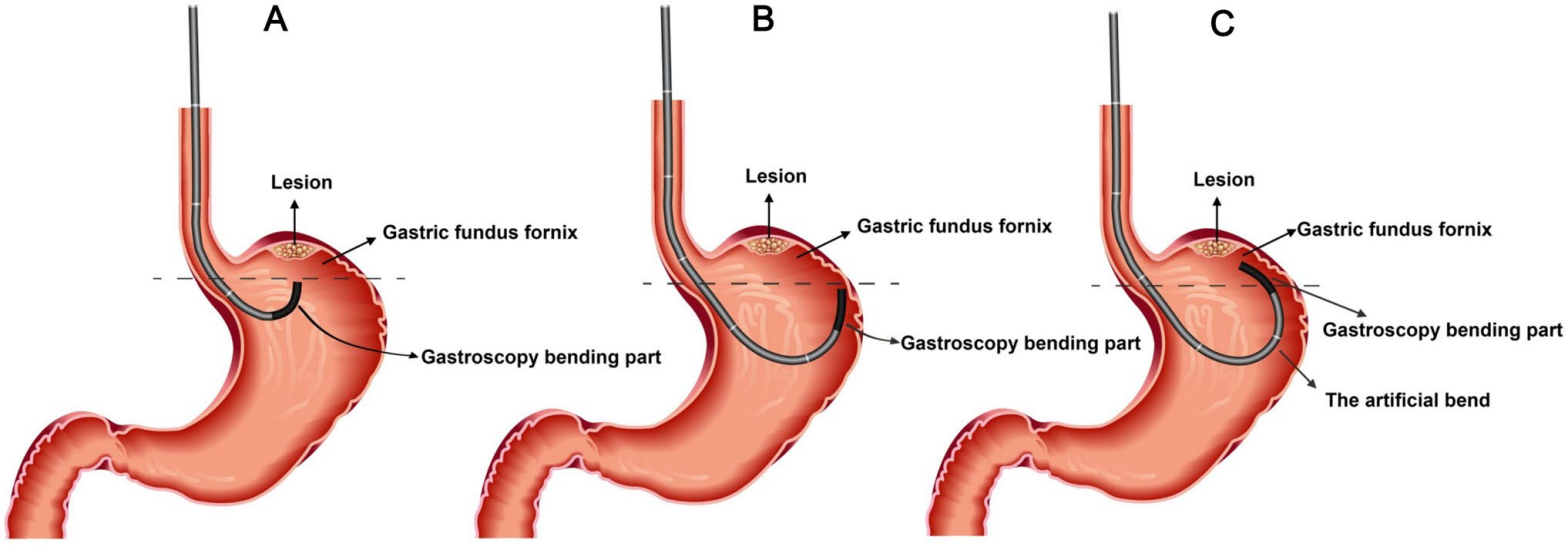
A diagram of DBM-ESD. **A** The bent portion of the gastroscope was reversed in the fundus. **B** The bent portion of the gastroscope touched the greater curvature of the upper segment of the gastric body. **C** The inserted portion of the gastroscope was bent at the upper segment of the gastric body, and together with the bent portion of the gastroscope, this constituted the DBM

## Patients and methods

### Subjects

In all, 190 patients with submucosal tumors in the gastric fundus who underwent endoscopic treatment at the Digestive Endoscopy Center of our hospital from March 2013 to February 2023 were retrospectively analyzed. This study was approved by our hospital’s ethics committee (Ethics Approval No. EC-2021-HS-001). All patients were informed about the technique and provided written consent before the procedure.

### Inclusion and exclusion criteria

The principal inclusion criteria were (1) lesions originating from the submucosa as confirmed by gastroscopy; (2) no evidence of lymph node involvement or distant metastasis; (3) agreement to undergo DBM-ESD or ESD treatment; and (4) discontinuation of antiplatelet agents or anticoagulants for more than 1 week. The exclusion criteria were (1) inability to tolerate anesthesia with tracheal intubation and (2) preexisting blood coagulation disorders.

## Methods

### Surgical instruments

The following instruments were used: Gastroscope (GIF-Q260J, Olympus (EG-580RD, FUJIFILM), Endoscopic Flushing Pump (OFP-2, Olympus), Carbon dioxide insufflator (UCR, Olympus), High-Frequency Electrosurgical Equipment (ICC 200D, ERBE), Distal Attachment (D-201-11804, Olympus), Injection Needle (INJ1- A1, Medwork), IT Knife (Single Use Electrosurgical Knife KD-611L, Olympus), IT Knife Nano (Single Use Electrosurgical Knife KD-612, Olympus), Dual Knife (KD650-L, Olympus), Coagulating forceps (Single Use Electrosurgical Forceps FD-410LR, Olympus), Ligating Device (SINGLE USE LIGATING DEVICE HX-400U-30, Olympus) (SINGLE USE LIGATING DEVICE LD-195, LeoMed, Changzhou, China), Disposable Ligating Loop (Loop-15, 20, 30, 40, LeoMed, Changzhou, China), Disposable Electrosurgical Snare (SD-240U-25, Olympus), clips (ROCC-D-26-230-C, Micro-tech, Nanjing, China. AG-51044-1950-135-16, AGS Medtech, Hangzhou, China. HX-610-135, Olympus).

## Procedure

### Double-bending method

**Fig. 2:**
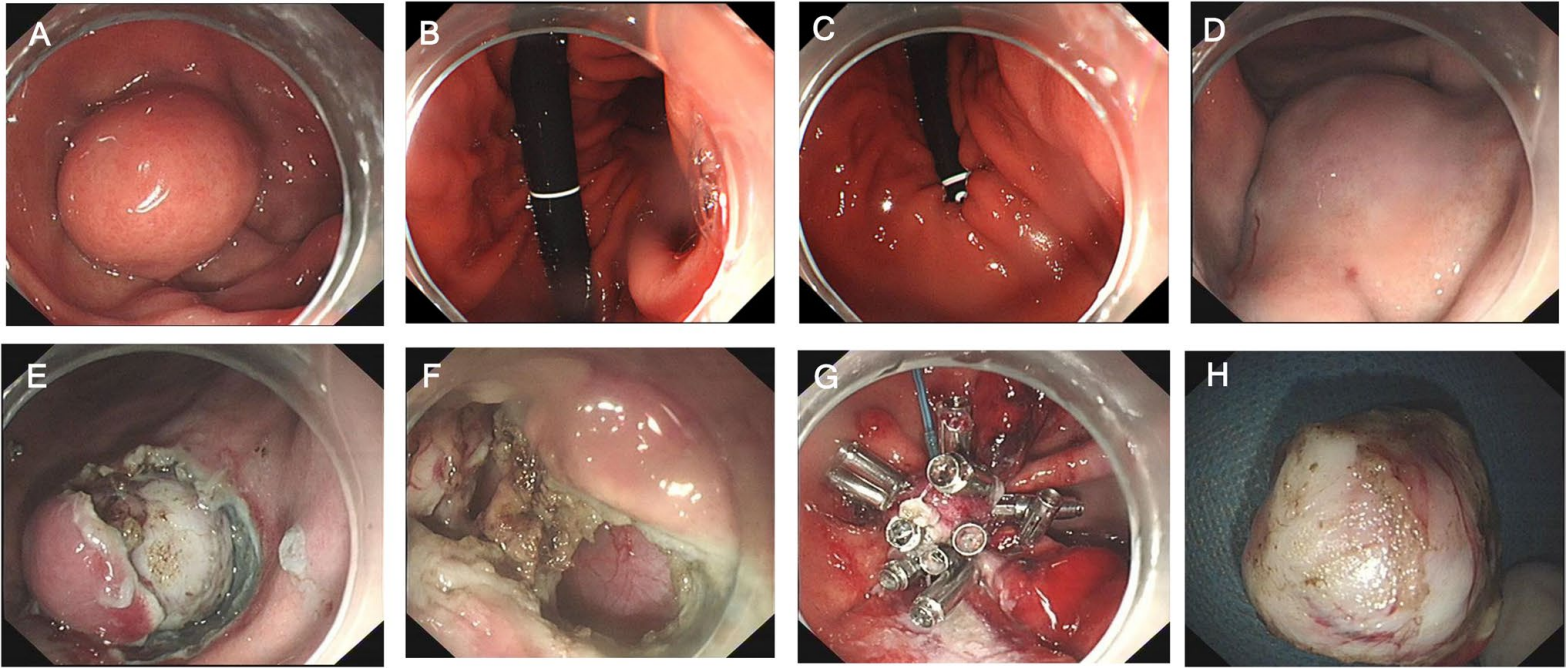
Representative gastroscopic view in patient. **A**. SMT was observed only via reversal of the bent portion of the gastroscope. **B**, **C**, and **D** The gastroscope body (**B**), the cardia (**C**), and the SMT (**D**) observed via the double-bending method. **E** View of the tumor during resection. **F** Gastric wall defects were found in the center of the wound surface. **G** The wound surface was closed. **H** View of the tumor after resection

After the gastroscope passed through the cardia, all of the gastric juice in the gastric cavity were routinely aspirated until the cavity was emptied. The stomach cavity was appropriately inflated, and the endoscope body was reversed in the fundus so that the fornix and the endoscope body could be observed via the gastroscope. The gas in the gastric cavity was aspirated until the body of the gastroscope touched the greater curvature of the upper part of the gastric body. The blocking force of the bent portion of the gastroscope against  the posterior wall and the greater curvature of the upper part of the gastric body was used as a support point so that the gastroscope could be pushed forward. Consequently, the inserted portion of the gastroscope was bent at the upper segment of the gastric body, and the inserted portion of the gastroscope could slide along the greater curvature of the upper segment of the gastric body, allowing access to the fornix of the gastric fundus. At this point, appropriate air injection exposed the fornix of the gastric fundus and the cardia. When the gastroscope was pushed forward, its tip moved toward the gastric fundus, and when the gastroscope was pulled backward, its tip moved away from the fundus. In this way, the gastroscope was able to easily approach the lesions in the fornix of the gastric fundus and around the cardia, with the body of the gastroscope parallel to the lesions. The gastroscope body was fixed in the upper gastric body by the stomach wall without the need for suspension (see Figs. 1, 2 and the video for details). The DBM was used for submucosal injection and submucosal dissection during the endoscopic treatment of SMTs in the fornix of the gastric fundus or near the cardia. After dissection, wound repair was performed via a combination of the DBM and conventional techniques.

### ESD procedures for gastric SMT

The patients underwent endotracheal intubation under intravenous anesthesia before endoscopic surgery. The dots around the lesion were marked with a dual knife. Saline containing methylene blue and sodium hyaluronate was injected into the submucosa. The mucosal and submucosal layers around the lesion were precut, after which the submucosal layer was dissected until the tumor was exposed. The tumor was then carefully dissected from the muscularis propria (MP) layer to ensure complete resection. If the tumor was located in the deep MP layer and close to the serosa, dissection away from the serosal layer would have been difficult; consequently, endoscopic full-thickness resection (EFR) was performed instead. In those cases, circumferential resection of the MP and serosa around the tumor was performed, and an “artificial” perforation was created. Coagulating forceps were used for adequate prophylactic hemostasis of the wound. Finally, clips were used to close the wound surface, and a gastric decompression tube was inserted. All the procedures were performed by three endoscopists (Zan Zuo, Qiang Guo, and Yu Zhang).

### Duration of the procedure

The procedure began from the time of the initial mucosal marking and ended when the last hemostasis clip was placed for both ESD and DBM-ESD.

### Postoperative management

Patients were asked to fast for 72 h. Antibiotics and proton pump inhibitors were routinely used to prevent infection and hemorrhage. Patients were observed for surgical complications, including bleeding, perforation, and infection. Oral proton pump inhibitors were prescribed for 8 weeks upon discharge from the hospital.

### Pathology

The tumor size and mitotic rate per 50 high-power fields (HPF) were determined. These criteria were used for GIST risk assessment, which was performed according to the National Institutes of Health consensus and classification [6].

### Follow-up

All patients were followed up with gastroscopy at 3, 6, and 12 months after the operation to observe wound healing.

### Statistical analyses

Statistical analyses were performed via IBM SPSS Statistics 18.0 (SPSS, Inc., Chicago, IL, USA). Categorical data are presented as numbers or percentages (%), whereas continuous data are expressed as means. Significant differences between groups were assessed via Student’s t test, while the chi-square test was applied to assess differences in proportions. Differences were considered significant at *P* < 0.05.

## Results

### Patient information

In all, 190 patients diagnosed with SMTs in the gastric fundus were included in this study (Table 1). DBM-ESD was performed in 100 patients (26 males and 74 females) with an average age of 55.04 ± 9.96 years and a lesion diameter of 1.98 ± 0.94 cm. ESD was performed in 90 patients (28 males and 62 females) with an average age of 50.80 ± 10.21 years and a lesion diameter of 1.79 ± 1.04 cm. Patients in the DBM-ESD group were older, on average, than those in the ESD group (*P* = 0.004). No significant difference was observed in tumor size between the two groups (*P* = 0.184).

**Table 1:** Clinical features of the patients in this study

	DBM-ESD	ESD	*P* value
Male/female	26/74	28/62	
Age (years)	55.04 ± 9.96	50.80 ± 10.21	*P* < 0.01
Tumor size (cm)			
Mean	1.98 ± 0.94	1.79 ± 1.04	0.18
Range	1–4	1–5	
Histological type			
GIST	90	88	
Leiomyoma	10	2	
Pathologic risk for GIST			
Very low risk	46(51.11)	57(64.77)	0.07
Low risk	44(48.89)	31(35.23)	
Mitotic index for GIST			
< 5/50HPF	90	88	1.00

*DBM-ESD* double-bending method ESD, *ESD* endoscopic submucosal dissection, *GIST* gastrointestinal stromal tumors

### Histopathology assessment

The histological type was GIST in 178 patients. Of the patients in the DBM-ESD group, forty-six were at very low risk, and 44 were at low risk. Of the patients in the ESD group, fifty-seven were at very low risk, and 31 were at low risk. No patient was at mild risk or high risk. No significant difference was observed in the proportion of very-low-risk and low-risk patients between the two groups. The mitotic index in all 178 patients was < 5 mitoses per 50 HPF (Table 1).

### Therapeutic outcomes and complications

The SMTs were completely resected in all patients. The R0 resection rate was 100% for both groups. The median procedure duration of the DBM-ESD group (54.2 ± 22.44 min) was significantly shorter than that of the ESD group (101.6 ± 65.26 min) (*P* < 0.001) (Table 2). The procedure time was compared across the three subgroups according to tumor size (Table 3). When the tumor diameter was ≤ 1 cm, the procedure durations in the DBM-ESD group and ESD group were 38.9 ± 8.74 min and 56 ± 20.95 min (*P* < 0.001), respectively. When the tumor diameter was > 1 cm and ≤ 2 cm, the durations in the DBM-ESD group and ESD group were 45.4 ± 21.09 min and 105.1 ± 51.13 min (*P* < 0.001), respectively. When the tumor diameter was > 2 cm, the durations in the DBM-ESD group and ESD group were 65.8 ± 21.78 min and 158.1 ± 65.3 min (*P* < 0.001), respectively. We found that when the tumor diameter exceeded 1 cm, the operation time in the ESD group was twice that of the DBM-ESD group, which indicates that dissection was faster for larger-diameter tumors with the DBM-ESD method. Together, these findings suggest that compared with ESD, DBM-ESD was more efficient in the treatment of submucosal tumors of the gastric fundus, especially in patients with large tumor diameters.

**Table 2:** Comparison of the resection efficiency and safety between the two groups

	DBM-ESD	ESD	*P* value
Cases	100	90	
R0 resection rate (%)	100	100	
Gastric wall defect	62(62)	47(52.22)	0.19
Mean duration of the procedure (min)	54.2 ± 22.44	101.6 ± 65.26	P < 0.01
Mean number of clips	12.60 ± 7.43	13.71 ± 8.29	0.33
Mean number of disposable ligating loop	0.78 ± 0.99	0.58 ± 0.72	0.11
Mean postoperative length of stay (days)	6.61 ± 1.45	6.98 ± 1.61	0.10
Postoperative complication			
Bleeding	1	0	1.00
Perforation	0	0	

*DBM-ESD* double-bending method ESD, *ESD* endoscopic submucosal dissection

**Table 3:** Comparison of the resection efficiency of the different subgroups

Subgroup (cm)	DBM	Cases	Tumor size (cm)		Duration of the procedure(min)	
			Mean	*P* value	Mean	*P* value
≤ 1	No	41	1 ± 0		56 ± 20.95	< 0.01
	Yes	28	1 ± 0		38.9 ± 8.74	
> 1, ≤ 2	No	17	1.45 ± 0.36	0.99	105.1 ± 51.13	< 0.01
	Yes	20	1.45 ± 0.29		45.4 ± 21.09	
> 2	No	32	2.99 ± 0.81	0.11	158.1 ± 65.3	< 0.01
	Yes	52	2.72 ± 0.69		65.8 ± 21.78	

*DBM* double-bending method

The percentages of patients who underwent “artificial” perforation operations in the DBM-ESD group and ESD group were 62% and 52.22%, respectively (*P* = 0.189) (Table 2). The mean numbers of clips used in the DBM-ESD group and the ESD group were 12.60 ± 7.43 and 13.71 ± 8.29, respectively (*P* = 0.331). The mean numbers of disposable ligating loops used in the DBM-ESD group and the ESD group were 0.78 ± 0.99 and 0.58 ± 0.72, respectively (*P* = 0.112). The mean postoperative lengths of hospital stay in the DBM-ESD group and the ESD group were 6.61 ± 1.45 and 6.98 ± 1.61, respectively (*P* = 0.100). One case of postoperative hemorrhage occurred in the DBM-ESD group, whereas no hemorrhage occurred in the ESD group (*P* = 1.000). Neither the DBM-ESD group nor the ESD group included any cases of postoperative perforation. All the patients’ wounds healed during the follow-up period.

## Discussion

The current treatments for gastric SMTs include endoscopic snare resection [7], endoscopic submucosal excavation (ESE) [8], submucosal tunneling endoscopic resection (STER) [9], and endoscopic full‑thickness resection (EFR) [10], the last three of which evolved from ESD. The gastric wall at the fornix is one of the thinnest areas of the stomach, and consequently, tumors in this area are difficult to treat endoscopically. Not only is the gastric wall thin in this region, but it is also difficult for the endoscope to reach this site. In addition, when the endoscope is inverted in this position, the direction of the endoscopic body movement is opposite to the direction of observation; moreover, the body of the endoscope is suspended in this position, which makes the device difficult to control. Consequently, the time required for endoscopic lesion resection, intraoperative hemostasis, and endoscopic repair of gastric wall perforation is significantly increased.

The method used in this study involved the removal of SMTs in the gastric fundus via modified traditional ESD, which is the same as the method reported by Guo [11]. However, the number of cases in that study was small, and the method was not compared with traditional methods. With DBM-ESD, the direction of endoscope body movement is the same as the direction of observation, and the endoscope body is not suspended in this position, which ensures the stability of the device. Moreover, the effects of respiration and large vessel pulsation on the procedure are reduced. This allows the operator to achieve smooth, convenient, complete endoscopic resection of the tumor, intraoperative hemostasis, and repair of the stomach perforation, which greatly shortens the operation time.

In our study, the duration of the endoscopic procedure in patients who underwent DBM-ESD was 54.2 ± 22.44 min, which was significantly shorter than that in patients who underwent ESD (101.6 ± 65.26 min). Moreover, a subgroup analysis revealed that the larger the tumor volume, the greater the advantage of DBM-ESD in terms of operation time. No significant differences were observed between the two groups in terms of the number of suture clips used, postoperative bleeding, perforation, or length of hospital stay. Compared with the conventional method, DBM-ESD can significantly shorten the procedure duration but does not increase the incidence of complications or prolong the length of hospital stay.

DBM procedures have been performed only in recent years, and after we identified that DBM-ESD was associated with significant advantages, most patients have since undergone surgeries using this method. However, in this study, all the surgeries were performed by three physicians who were proficient in ESD. Therefore, temporal bias and operator learning curve effects did not significantly influence the results. Moreover, we identified several limitations of this method. First, DBM-ESD cannot be performed in every patient. In this study, DBM-ESD was not successful in a few patients because of the presence of a wide gastric cavity in the fundus and upper gastric body. Second, the left and right movements of the endoscope body and the visual field adjustment were also affected, and adjustments of the visual field by repeated insertion and withdrawal of the endoscope, changes to the gas volume in the gastric cavity, and adjustments of the small knob were necessary. Third, our study was a retrospective analysis; therefore, a randomized control study is still needed to further confirm our findings.

In conclusion, DBM-ESD can significantly shorten the surgical time needed to resect gastric fundal SMTs. DBM-ESD is a better choice for patients whose tumors are too large for conventional endoscopic resection.

## Supplementary Information

Below is the link to the electronic supplementary material.

Supplementary file1 (MP4 12882 KB)
